# Comparison of telbivudine and entecavir on the change of off- treatment eGFR after 3 years of treatment in non-cirrhotic chronic hepatitis B patients

**DOI:** 10.1186/s12876-017-0582-0

**Published:** 2017-01-31

**Authors:** Yang-Sheng Lin, Shou-Chuan Shih, Horng-Yuan Wang, Ching-Chung Lin, Chen-Wang Chang, Ming-Jen Chen

**Affiliations:** 1 Division of Gastroenterology, Department of Internal Medicine, MacKay Memorial Hospital, Taipei Campus, No. 92, Sec. 2, Chungshan North Road, 104 Taipei, Taiwan; 2 MacKay Junior College of Medicine, Nursing, and Management, Taipei, Taiwan; 3 MacKay Medical College, New Taipei City, Taiwan

**Keywords:** Telbivudine, Entecavir, Chronic hepatitis B, Off-treatment, Renal function, eGFR

## Abstract

**Background:**

The change of estimated glomerular filtration rate (eGFR) with off-treatment nucleos(t)ide analogues (NA) in chronic hepatitis B patients (CHB) is unclear. This study is aimed to evaluate the off-treatment eGFR after 3 years of therapy with telbivudine (LdT) or entecavir (ETV) and to assess predictive factors for eGFR improvement.

**Methods:**

From January 2009 to December 2011, we identified NA-naïve patients who were at least 20 years of age diagnosed with compensated CHB. All patients received a 3-year NA treatment and 1 year off-treatment follow-up; the initial selection of patients for LdT or ETV treatment was at the physicians’ discretion. An increase of more than 10% in eGFR from the baseline was identified as an improvement. The change of chronic kidney disease stages were recorded and compared with baseline at year 3 and year 4, respectively.

**Results:**

This study included two groups consisting of 46 patients each (each with3 years of treatment with LdT or ETV). In LdT-treated patients, the mean eGFR increased from 94.3 ± 28.3 to 104.0 ± 31.2 mL/min/1.73 m^2^ in year 3 (*p* = 0.01) and from 104.0 ± 31.2 to 104.0 ± 28.8 mL/min/1.73 m^2^ in year 4 (*p* = 0.99). However, in ETV-treated patients, the mean eGFR decreased from 93.1 ± 26.1 to 85.5 ± 25.1 mL/min/1.73 m^2^ in year 3 (*p* = 0.0009) and from 85.5 ± 25.1 to 87.7 ± 24.8 mL/min/1.73 m^2^ in year 4 (*p* = 0.2). After a multivariate analysis, the predictors for the off-treatment eGFR improvement were the LdT treatment (odds ratio [OR], 3.97 (1.37–11.5), *p* = 0.01) and pre-treated eGFR (OR, 0.98 (0.95–1.00), *p* = 0.04).

**Conclusions:**

At year 4, 48.8 and 21.3% patients had an improved eGFR from baseline in LdT and ETV patients, respectively. Telbivudine may have a protective renal effect that can last for one year after treatment in non-cirrhotic CHB patients without a virological breakthrough.

## Background

Nucleos(t)ide analogs (NA) are widely used for the treatment of hepatitis B and have been shown to reverse fibrosis and cirrhosis, and reduce the risk of hepatic decompensation and hepatocellular carcinoma [1, 2]. Although NAs are effective in suppressing hepatitis B virus (HBV) replication, most patients require long-term treatment. Safety is one of the major considerations with a long-term and even life-long use of NAs and needs to gain more attention [3]. In particular, the effects of HBV infection or treatment agents on the renal dysfunction in CHB patients require further studies.

There are currently 5 oral antiviral agents for CHB: 2 nucleotides (adefovir and tenofovir) and 3 nucleosides (lamivudine, entecavir (ETV), and telbivudine (LdT)). All of these oral antiviral agents are primarily eliminated unchanged through the renal route, therefore, can have an effect on the mitochondrial or proximal tubular microstructures. The nephrotoxic potential seems to be more prevalent in nucleotides (adefovir and tenofovir) [3]. The exact mechanism is largely unknown and is likely to involve multiple pathophysiological pathways [3]. From a review article, during long-term therapy, minimal rates of eGFR decline have been reported, except for LdT [4]. Furthermore, retrospective analyses of LdT have demonstrated a potential renal improvement in a broad spectrum of CHB patients. In a large cohort of patients with CHB receiving LdT, the estimated GFR (eGFR) [using MDRD formula] increased by 14.9 mL/min or by 16.6% from baseline to year 4 [5].

This improvement in eGFR was more evident in the LdT-treated patients with stage 2 chronic kidney disease (i.e. eGFR 60–90 mL/min), wherein, 74% of the patients regained normal renal function (i.e. eGFR >90 mL/min) after 4 years of treatment [6]. This effect was maintained during long-term therapy and was also observed in patients after 2 years off treatment [6]. The mechanism of the beneficial effect of LdT therapy on renal function is still undetermined.

It is still unknown if the improvement in renal function is specific to LdT or what is the result of the maintenance after LdT off-treatment? As a hyperendemic area of CKD of 11.9% in general population [7], choice of NA with the potential renoprotection after off-treatment follow up is intriguing. In Taiwan, the Bureau of National Health Insurance reimburses NA treatment for up to 3 years in treatment-naïve CHB patients if there is no evidence of virological breakthrough during the treatment period. To the best of our knowledge, there is no published study comparing the effects of LdT and ETV on renal function after the treatment.

## Methods

### Patients

From January 2009 to December 2011, we identified NA-naïve patients who were at least 20 years of age and diagnosed with compensated CHB. All patients received a 3-year NA treatment and had a 1-year follow-up after off treatment; the initial selection of patients for LdT or ETV treatment was at the physicians’ discretion. According to the current guidelines, antiviral treatment is recommended for HBeAg-negative patients with a HBV-DNA level over 2000 IU/mL and serum alanine transaminase (ALT) >2 x upper normal limit (ULN) for 3 months; all HBeAg-positive patients with HBV-DNA level over 20,000 IU/mL and serum ALT above >2 x ULN or any HBeAg-positive patients with ALT >5 x ULN).

We investigated the underlying diseases in the beginning of treatment including chronic kidney disease stages, hypertension, diabetes and prescribed potential renal toxicity agents such as nonsteroidal anti-inflammatory drugs (NSAIDs) or immunosuppressive agents. The patients were regularly followed up with abdominal sonography and serum HBV-DNA every 6 months during the treatment, and serum liver function tests and eGFR every 3 months for one year after the treatment according to the national guideline. If there were two more tests during the 3-month interval, we chose the test more closely to their respective check points (such as at year 3 or year 4).

The patients with coexisting HIV or HCV infection, prior NA therapy, liver cirrhosis or acute fulminant hepatitis, autoimmune disease, and malignancy were excluded from the study. The appropriate dosages (or dosing interval) of LdT and ETV were adjusted according to the patient’s renal function. The study program was approved by the Institutional Review Board of MacKay Memorial Hospital (15MMH-ISO-014).

### Assessments

Baseline data of patients were retrieved from the medical records that included age, sex, HBV DNA level (IU/mL), hepatitis B surface antigen/anti-HBs, hepatitis B e-antigen /anti-HBe, levels of ALT, albumin, total bilirubin and creatinine, and initial NA treatment. The renal function was estimated based on MDRD calculation for eGFR (mL/min/1.73 m^2^): 186 x creatinine (mg/dL)^−1.154^ x age^−0.203^ x 0.742 (if female). An eGFR more than 90 mL/min/1.73 m^2^ was classified as chronic kidney disease 1 (CKD stage 1). An eGFR of 60–89 mL/min/1.73 m^2^ was classified as CKD stage 2. An eGFR of 30–59 mL/min/1.73 m^2^ was classified as CKD stage 3. An eGFR of 15–29 mL/min/1.73 m^2^ was classified as chronic kidney disease CKD stage 4.

As compared to the baseline, patients with an increasing eGFR >10% was seen as an improvement. The number of patient changing CKD stages compared with baseline at year 3 and year 4 were recorded in each groups. Factors such as age, sex, NA, HBV DNA, HBeAg status, and baseline eGFR were evaluated to predict improvement in eGFR at year 3 and year 4, respectively.

### Statistical analysis

Continuous variables were expressed as mean ± standard deviation (SD) and compared using the Student’s *t*-test. Categorical data were compared using the Fisher’s exact test or *χ*2 test, as appropriate. Continuous variables that were not normally distributed were evaluated using Mann-Whitney’s *U*-test. We used SPSS version 12.0 (SPSS Inc., Chicago, IL, USA) for all statistical analyses. All statistical tests were two-tailed, and *P* <0.05 was considered statistically significant. We included all the covariates for the logistic regression analysis and a multivariate analysis was also performed to assess the predictive factors.

## Results

### The demographics and characteristics of 92 HBV patients assigned to treat with LdT or ETV

A total of 180 CHB patients were enrolled in this retrospective study, of which 89 patients were treated with LdT and 91 patients were treated with ETV according to the Taiwanese Health Insurance Guidelines. The initial selection of NA for treatment was at the physicians’ discretion. Nineteen patients in the LdT group had a virological breakthrough during the 3-year treatment period, 15 patients decided to continue the treatment with self-paid medicine, and 9 patients had a viral breakthrough within 1 year after treatment following 3 years of therapy. Two patients in the ETV group changed to another NA due to headache and intent to become pregnant, 38 patients decided to continue the treatment with self-paid medicine, and 5 patients experienced viral breakthrough within 1 year after the treatment.

**Fig. 1:**
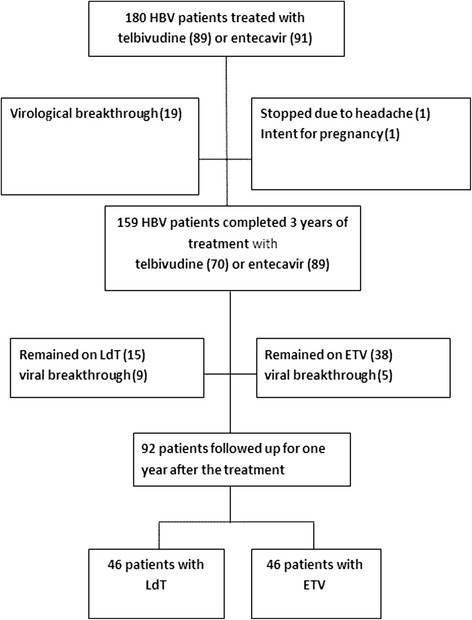
Flow chart of the treatment

In total, 2 groups of 46 patients with HBV treated with LdT or ETV, respectively, were analyzed in this study (Fig. 1). The dosages and dosing interval were adjusted according to the patient’s renal function. Only one patient in the ETV group modified the dosing interval every two days according to his creatinine clearance (25 mL/min), which was less than 50 mL/min.

The mean age of patients who completed 3 years of treatment with LdT and ETV were 46.3 and 51.3 years, respectively. The baseline demographic data were balanced at baseline for age, sex, comorbidity of chronic kidney disease stages, hypertension, and diabetes and prescribed with potential renal toxicity agents (Table 1). There were 63% (29 of 46) and 60.1% (28 of 46) HBeAg-positive patients in the LdT-treated group and ETV-treated groups, respectively (*p* = 0.57). Nonsteroidal anti-inflammatory drugs (NSAIDs) were used in 2.2% of the ETV group and 8.8% in the LdT group; immunosuppressive agents were used in 2.2% of the ETV group and 2.2% in the LdT group.

**Table 1:** The demographics and change of CKD stages of 92 HBV patients assigned to be treated with LdT or ETV

	LdT (*n* = 46)	ETV (*n* = 46)	*P*-value
Age (mean ± SD)	46.3 ± 13.4	51.3 ± 11.5	0.06
Sex (M) (%)	35 (76.1)	35 (76.1)	1
CKD stages (%)			0.31
CKD 1	23 (50.0)	20 (43.5)	
CKD 2	19 (41.3)	24 (52.2)	
CKD 3	4 (8.7)	1 (2.2)	
CKD 4	0 (0)	1 (2.2)	
Diabetes (%)	11 (23.9)	8 (17.4)	0.44
Hypertension (%)	15 (32.6)	8 (17.4)	0.09
Nephrotoxic agents (%)	13 (28.3)	10 (21.7)	0.47
Cyclophosphamide (%)	1 (2.2%)	1 (2.2%)	
NSAID (%)	4 (8.8%)	1 (2.2%)	
HBV DNA (log IU/mL) (IQR)	6.6 (1.9)	6.2 (2.5)	0.93
HBe Ag (+) (%)	29 (63.0)	28 (60.1)	0.57
eGFR (± SD) (mL/min/1.73 m^2^)			
Baseline	94.3 ± 28.3	93.1 ± 26.1	0.87
Year 3	104.0 ± 31.2	85.5 ± 25.1	0.005
Year 4	104.0 ± 28.8	87.7 ± 24.8	0.04

The age and eGFR were analyzed with Student’s *t*-test. The sex, diabetes, hypertension and nephrotoxic agents, HBeAg status and CKD stages were analyzed with *χ*2 test and the others were analyzed with Mann-Whitney’s *U*-test

### The change of eGFR at the end of 3 years of treatment with LdT and ETV compared with baseline

**Fig. 2:**
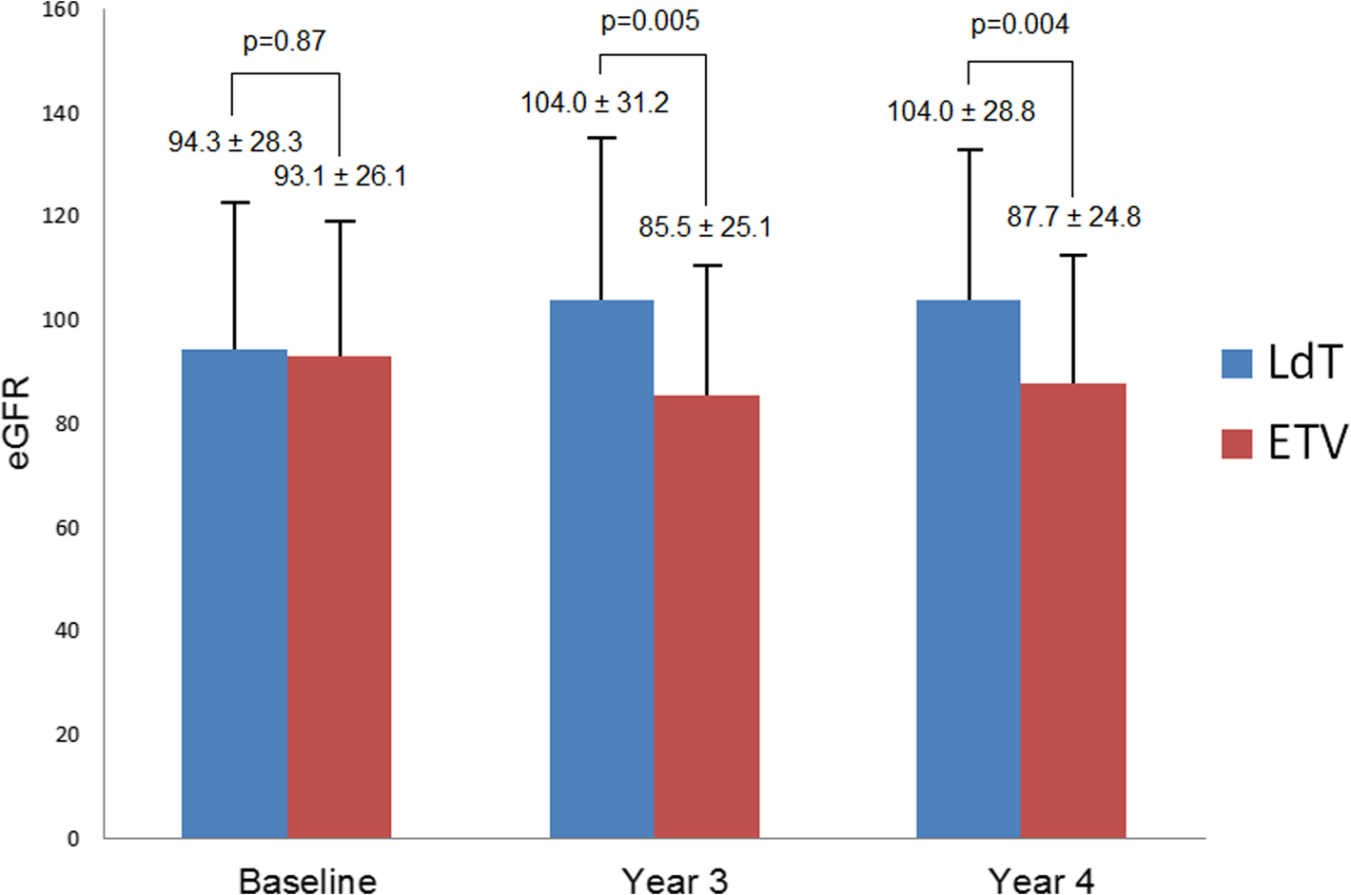
Change of eGFR at the end of 3-years of treatment and 1 year after the treatment in LdT and ETV groups compared to the baseline

At the end of year 3, mean eGFR increased from 94.3 ± 28.3 to 104.0 ± 31.2 (mL/min/1.73 m^2^) (*p* = 0.01) in LdT-treated patients, whereas it decreased from 93.1 ± 26.1 to 85.5 ± 25.1 (mL/min/1.73 m2) (*p* = 0.0009) in ETV-treated patients (Fig. 2). In addition, 23 of 46 (50.0%) patients had 10% more eGFR improvement compared to baseline in LdT patients. Similarly, 6 of 46 patients (13.1%) showed an improvement in the ETV group.

Eleven patients improved their CKD stages (8 patients from stage 2 to stage 1 and 3 patients from stage 3 to stage 2) and 2 patients worsened their CKD stages (one from stage 2 to stage 3 and one from stage 1 to stage 2) in the LdT group, one patient improved his CKD stage from stage 2 to stage 1 and 8 patients worsened their CKD stages (7 patients from stage 1 to stage 2 and one patient from stage 2 to stage 3) in the ETV group at year 3, compared to the baseline, respectively (*p* = 0.004) (Table 2).

**Table 2:** The change of CKD stages compared with baseline at year 3 and year 4 with LdT or ETV

	LdT (*n* = 46)	ETV (*n* = 46)	*P*-value
Year 3 compared with baseline			
CKD stages (%)			0.004
CKD improved	11 (24.0)	1 (2.2)	
CKD no changed	32 (69.6)	37 (80.4)	
CKD worsen	3 (6.5)	8 (17.4)	
Year 4 compared with baseline			
CKD stages (%)			0.07
CKD improved	11 (23.9)	5 (10.9)	
CKD no changed	32 (69.6)	32 (69.6)	
CKD worsen	3 (6.5)	9 (19.6)	

The CKD status was analyzed with *χ*2 test

After a multivariate analysis of patients’ age, sex, pre-treatment eGFR, HBeAg status, HBV DNA levels, NA, and nephrotoxic agents, the predictors for an eGFR improvement at the end of 3-year were the LdT treatment (odds ratio [OR], 7.97 [2.46–25.7], *p* = 0.001) (Table 3).

**Table 3:** The predictors for eGFR improvement at the end of 3-years of treatment with LdT and ETV compared with baseline

	Univariate logistic regression			Multivariate logistic regression		
	OR	95% CI	*p*-value	OR	95% CI	*p*-value
Age	0.99	0.96–1.03	0.75	0.98	0.94–1.03	0.48
Sex	0.77	0.26–2.22	0.62	1.09	0.28–4.21	0.90
LdT	6.67	2.37–18.8	0.0001	7.97	2.47–25.7	0.001
HBV DNA	1	1	0.83	1	1	0.26
HBeAg	0.50	0.20–1.27	0.13	0.33	0.09–1.24	0.10
Baseline eGFR	0.98	0.96–1.00	0.067	0.98	0.96–1.00	0.08
Nephrotoxic agents	1.70	0.36–8.15	0.51	1.15	0.17–7.61	0.89

### The change of eGFR 1 year after the off-treatment period compared with baselines

The present analyses focused on key therapeutic endpoints at 1 year after the off-treatment period. During the fourth year without viral breakthrough, the eGFR increased from 94.3 ± 28.3 to 104.0 ± 28.8 mL/min/1.73 m^2^ in LdT patients and decreased from 85.5 ± 25.1 mL/min/1.73 m2 to 87.7 ± 24.8 mL/min/1.73 m^2^ in ETV patients (Fig. 2). Overall, 21 of 46 (48.8%) patients in the LdT group and 10 of 46 (21.3%) in the ETV group showed an improved renal function 1 year after the treatment compared with baseline. Eleven patients improved their CKD stages (8 patients from stage 2 to stage 1 and 3 patients from stage 3 to stage 2) and 2 patients worsened their CKD stages (1 from stage 2 to stage 3 and 1 from stage 1 to stage 2) in the LdT group, 4 patients improved their CKD stages (1 patient from stage 3 to stage 2 and 3 patients from stage 2 to stage 1) and 9 patients worsened their CKD stages from stage 1 to stage 2 in the ETV group at year 4, compared to the baseline, respectively (*p* = 0.07) (Table 2). There was no significant change in eGFR from the 3^rd^ to the 4^th^ year of treatment in the LdT group (104.0 – 104.0 mL/min/1.73 m^2^, *p* = 0.99) and ETV group (85.5 ± 25.1 – 87.7 mL/min/1.73 m^2^, *p* = 0.2). After a multivariate analysis, the predictors for off-treatment eGFR improvement were the LdT treatment (OR, 3.97 (1.37–11.5), *p* = 0.01) and pre-treated eGFR (OR, 0.98 [0.95–1.00], *p* = 0.04) (Table 4).

**Table 4:** The predictors for eGFR improvement 1 year after offtreatment with LdT and ETV compared with baseline

	Univariate logistic regression			Multivariate logistic regression		
	OR	95%CI	*P*-value	OR	95%CI	*P*-value
Age	1.03	0.99–1.07	0.10	1.03	0.98–1.08	0.20
Sex	0.50	0.16–1.51	0.22	0.86	0.23–3.19	0.82
LdT	3.02	1.22–7.51	0.02	3.97	1.37–11.5	0.01
HBV DNA	1	1	0.50	1	1	0.70
HBeAg	0.43	0.17–1.10	0.08	0.64	0.19–2.15	0.47
Baseline eGFR	0.98	0.96–1.00	0.03	0.98	0.95–1.00	0.04
Nephrotoxic agents	1.53	0.32–7.29	0.60	1.04	0.17–11.0	0.97

## Discussion

Nucleos(t)ides are generally safe and well-tolerated, but side effects have been reported including nephrotoxicity, neuropathy, myopathy, lactic acidosis, and a decrease in bone mineral density. Of these, nephrotoxicity associated with nucleotide treatment has received the most attention. The GLOBE trial, which was composed of about 65% Asian patients treated with LdT, demonstrated the safety profile of renal function [5]. The renal function measured by eGFR increased from baseline and remained the same during continuous treatment for up to 6 years and also in the off-treatment period [6]. However, the promising results could not be extrapolated to a wider spectrum of patients in clinical and real-life practice. Post-marketing real-life observational studies are needed to demonstrate the effectiveness of these agents in different regions. The mechanism responsible for the change of renal function during long-term NA treatment is still under investigation and the effect of maintenance after the off-treatment follow-up is unknown. To the best of our knowledge, there is no published study comparing the effects of LdT and ETV on off-treatment renal function.

This study aimed to compare LdT versus ETV off-treatment effects on the changes in eGFR. Entecavir is very potent and maintains a high genetic barrier to HBV resistance, and thus, is widely prescribed. As inferred from the literature, ETV was highly effective in suppressing the HBV DNA replication at undetectable levels and had a very low resistance rate (1.2%) in NA-naive HBeAg-negative patients for up to 5 years [7]. Telbivudine has renal protection potential but is less potent compared to ETV. The rationale of comparing LdT to ETV is that we want to know whether the potential renal protective effect is dependent on its antiviral effect. A question remains as to why tenofovir is not chosen for treatment. This is because some patients with potential renal risk or renal dysfunction at baseline were excluded from tenofovir clinically due to its renal toxicity [3].

All international guidelines indicate that the primary goal of CHB treatment is to permanently suppress the HBV replication to anti-HBe seroconversion, HBsAg loss, or anti-HBs seroconversion with undetectable HBV DNA [1, 2, 8]. However, in Taiwan, the health insurance reimburses NA treatment for up to 3 years in patients with naïve CHB if there is no virological breakthrough during the treatment period regardless of meeting the treatment stopping criteria because of medical resource constraints. This restricted therapy policy allows us to select a relatively homogenous CHB patients (viremia <0^6^ for HBeAg negative or <10^8^ for HBeAg positive) treating group according to the national guideline with a defined (3-year) treatment period to evaluate the change in eGFR after off-treatment.

After 1 year off-treatment, their eGFR remained stable, as seen during the end of 3-year therapy. In LdT-treated patients, the mean eGFR increased from 94.3 to 104.0 mL/min/1.73 m2 in year 4. However, in ETV-treated patients, the mean eGFR decreased from 93.1 to 87.7 mL/min/1.73 m^2^ in year 4. In the fourth year, 48.8 and 21.3% patients had an improved eGFR from the baseline in LdT-treated and ETV-treated patients, respectively. The benefit of LdT on the off-treatment eGFR improvement is unique from that of ETV because the effect is not related to viral suppression. It also indicates that the increase in eGFR was influenced by LdT itself rather than the control of HBV infection. Chan et al. suggested that the direct mechanism responsible for the improvement of renal function could be the increased blood flow by LdT that improves the tubular dysfunction [9]. A possible effect of LdT could be on the kidney structures or inflammatory/fibrotic pathways. The mechanisms of NA excretion of the kidney should also be explored by studying the expression of transport pumps (e.g., hOAT1, hOAT3, MRP4) in cells of the proximal tubules [10, 11]. A previous study reported that the LdT treatment can affect the angiotensin-converting enzyme that can control the renin-angiotensin aldosterone regulatory system. However, further investigations are required to study this phenomenon under the pharmaco-pathophysiology [12]

In some real-life cohort studies, long-term LdT therapy resulted in an improved eGFR, while ETV therapy did not significantly influence eGFR [13–16]. However, in our study, ETV group showed a slight decline of eGFR in year 3 and during the maintenance effect 1 year after treatment. This may be attributed to the small sample size or selection biases.

Multivariate analysis of baseline factors in the GLOBE study that predicted a shift in eGFR from baseline of 60–90 to 90 mL/min/1.73 m^2^ in year 2 were the LdT treatment (OR, 2.51), younger age (OR, 0.94), and non-Caucasian race (OR, 0.34). In our study, we found similar results. A shift in eGFR from baseline of CKD stage 2 (60–90 mL/min/1.73 m^2^) to CKD stage 1 (>90 mL/min/1.73 m^2^) in year 3 was 8 of 19 (42%) patients in LdT group and 1 of 24 (4.1%) in ETV group. Multivariate analysis of baseline viral or host factors in our study that predicted a maintenance of eGFR improvement in year 4 were LdT treatment (OR, 3.97 (1.37–11.5) *p* = 0.01) and insufficient pre-treated eGFR (OR, 0.98 (0.95–1.00), *p* = 0.04), which is similar to previous studies [17, 18]. The lack of association between the change in eGFR and on-treatment virologic or serologic response would support a direct beneficial effect on the kidneys rather than an indirect effect from the HBV suppression [17].

The improvement of glomerular filtration is particularly important in patients with a slightly abnormal eGFR at baseline and could have important clinical implications. In our study, 11 patients improved their CKD stages and 2 patients worsened their CKD stages in the LdT group; one patient improved his CKD stage and 8 patients worsened their CKD stages in the ETV group at year 3 compared to baseline, respectively. (*p* = 0.004). Nevertheless, several concerns have been raised regarding the potential use of LdT as the first-line NA in CHB. Although its use has been associated with an improvement of renal function, it remains a low genetic barrier NA to overcome drug resistance in CHB patients and neuromuscular adverse effects [18].

There are many limitations in our study. First, there was a small sample size, retrospective design, and selection bias in choosing the initial medication. Second, it is still unclear the optimum level of eGFR change considered as clinically significant. Recent evidence suggests that a 30% decline of eGFR over 2 years is strongly and consistently associated with the risk of end-stage renal disease and mortality [19]. However, we do not know how much improvement is clinically relevant. Most clinical studies use eGFR >10% as a standard, although this needs to be further confirmed. Finally, we have only described the differences in the eGFR between the 2 treated groups to hypotheses a direct influence on eGFR without a relevant pathophysiologic theory. Clinical trials with a large sample size, randomized controlled design, dose dependence analysis, and long-term follow-up period are needed to confirm our findings.

## Conclusions

The mechanism behind the potential renal protective effect of LdT is still unclear, although it appears to be independent of its antiviral effect on HBV. Our findings show that LdT may have a protective renal effect that can last for one year after the treatment in selected non-cirrhotic CHB patients without a virological breakthrough.

## Abbreviations

ALT: Alanine transaminase
CHB: Chronic hepatitis B patients
CKD: Chronic kidney disease
eGFR: Estimated glomerular filtration rate
ETV: Entecavir
HBV: Hepatitis B virus
LdT: Telbivudine
NA: Nucleos(t)ide analogs
NSAIDs: Nonsteroidal anti-inflammatory drugs
